# *Agaricus meleagris* pyranose dehydrogenase: Influence of covalent FAD linkage on catalysis and stability

**DOI:** 10.1016/j.abb.2014.07.008

**Published:** 2014-09-15

**Authors:** Iris Krondorfer, Dagmar Brugger, Regina Paukner, Stefan Scheiblbrandner, Katharina F. Pirker, Stefan Hofbauer, Paul G. Furtmüller, Christian Obinger, Dietmar Haltrich, Clemens K. Peterbauer

**Affiliations:** a Department of Food Science and Technology, Food Biotechnology Laboratory, BOKU – University of Natural Resources and Life Sciences, Muthgasse 11, 1190 Vienna, Austria; b Department of Chemistry, Division of Biochemistry, BOKU – University of Natural Resources and Life Sciences, Muthgasse 18, 1190 Vienna, Austria

**Keywords:** Pyranose dehydrogenase, Covalent flavinylation, FAD, Thermal stability, Conformational stability, Semiquinone radical

## Abstract

Pyranose dehydrogenase (PDH) is a monomeric flavoprotein belonging to the glucose–methanol–choline (GMC) family of oxidoreductases. It catalyzes the oxidation of free, non-phosphorylated sugars to the corresponding keto sugars. The enzyme harbors an FAD cofactor that is covalently attached to histidine 103 via an 8α-N(3) histidyl linkage. Our previous work showed that variant H103Y was still able to bind FAD (non-covalently) and perform catalysis but steady-state kinetic parameters for several substrates were negatively affected. In order to investigate the impact of the covalent FAD attachment in *Agaricus meleagris* PDH in more detail, pre-steady-state kinetics, reduction potential and stability of the variant H103Y in comparison to the wild-type enzyme were probed. Stopped-flow analysis revealed that the mutation slowed down the reductive half-reaction by around three orders of magnitude whereas the oxidative half-reaction was affected only to a minor degree. This was reflected by a decrease in the standard reduction potential of variant H103Y compared to the wild-type protein. The existence of an anionic semiquinone radical in the resting state of both the wild-type and variant H103Y was demonstrated using electron paramagnetic resonance (EPR) spectroscopy and suggested a higher mobility of the cofactor in the variant H103Y. Unfolding studies showed significant negative effects of the disruption of the covalent bond on thermal and conformational stability. The results are discussed with respect to the role of covalently bound FAD in catalysis and stability.

## Highlights

• The mutation H103Y slowed down the reductive half-reaction by three orders of magnitude.
• Secondary structure composition was not altered according to CD spectra.
• EPR spectroscopy identified a semiquinone radical in the wild-type and variant H103Y.
• Thermal and conformational stability was negatively affected by the mutation.

## Introduction

Pyranose dehydrogenase (PDH, EC 1.1.99.29)^1^ is a monomeric FAD-dependent oxidoreductase and member of the glucose–methanol–choline oxidase (GMC) family. The glycoprotein is around 75 kDa in size and was first isolated from the edible basidiomycete *Agaricus bisporus* [1]. It catalyzes the oxidation of free, non-phosphorylated sugars at different C-atoms to the corresponding keto-derivatives. PDH is unable to utilize oxygen, suitable electron acceptors include ferrocenium hexafluorophosphate, ABTS^+•^, 1,4-benzoquinone or 2,6-dichloroindophenol (DCIP). The biological role of the enzyme is not entirely clear, a participation in lignocellulose degradation or the defense against antimicrobial substances produced by plants seem plausible [2].

An estimated 10% of all flavoproteins carry a covalently bound cofactor [3]. Pyranose dehydrogenase is one of these rare examples, like the closely related enzyme pyranose 2-oxidase (POx). The two proteins possess the most common type of covalent FAD-linkage, the 8α-histidyl-FAD, connected via the N3 of histidine 103 (PDH) and 167 (POx) [4], [5]. Another possibility of a covalent FAD-linkage, 8α-S-cysteinyl-FAD, is not very widespread. The extremely rare 8α-O-tyrosyl linkage was only found in *p*-cresol methylhydroxylase and 4-ethylphenol methylene hydroxylase [6], [7]. A double-covalent linkage, the 8α-N1-histidyl-6-S-cysteinyl-FAD, was first described in 2005 in the enzyme glucooligosaccharide oxidase [8]. All bicovalent flavoproteins characterized so far belong to the so-called VAO (vanillyl-alcohol-oxidase) family [9].

The formation of the FAD-linkage is believed to mostly occur as an autocatalytic post-translational reaction. The covalent incorporation of the FAD cofactor seems to be possible after folding of the protein in a lock-and-key manner, as shown in VAO and monomeric sarcosine oxidase (MSOX) [10], [11], [12]. Recently, a protein was discovered which is required for the covalent flavinylation of a bacterial flavoprotein controlling succinate dehydrogenase activity [13]. By looking at the fold or the sequence of a flavoprotein, one cannot predict a covalent or non-covalent linkage. Interestingly, the majority of flavoproteins carrying a covalently linked cofactor are oxidases, which may be explained by the higher reduction potential due to the covalent linkage and the resulting limitation of suitable electron acceptors other than dioxygen [3]. However, PDH as a flavoprotein dehydrogenase with a covalently linked cofactor and a very high midpoint potential of +150 mV seems to be an exception to this observation [14].

The removal of the covalent FAD-bond in POx, also a member of the GMC flavoprotein family, resulted in a tightly, but non-covalently bound FAD. Variant H167A showed a decreased turnover rate and lower reduction potential, but no significant structural changes were observed [15]. *A. meleagris* PDH variant H103Y was recently found in a screening of twelve site-saturation mutagenesis libraries of amino acids around the active site to show increased oxygen reactivity. The FAD-cofactor was shown to be bound in a tight, but non-covalent manner. Steady-state kinetics demonstrated that the mutation decreased the catalytic efficiencies towards sugar-substrates as well as electron acceptors by around one order of magnitude [16].

In this study, the PDH variant lacking the covalently linked FAD-cofactor (H103Y) was used to investigate the effect of the non-covalent linkage on catalysis and stability of the protein in more detail. Applied methods included electron paramagnetic resonance (EPR)-, stopped-flow UV–vis- and electronic circular dichroism (ECD)-spectroscopy as well as unfolding experiments by using fluorescence spectroscopy and differential scanning calorimetry (DSC).

## Materials and methods

### Materials

All chemicals were purchased from Sigma Aldrich (St. Louis, MO), Roth (Karlsruhe, Germany) or VWR (Radnor, PA). *Pichia pastoris* strain X-33 and Zeocin-resistance encoding shuttle vector pPICZB were from Invitrogen (Carlsbad, CA). *Escherichia coli* strain NEB5α was from New England Biolabs (Ipswich, MA).

### Site-directed mutagenesis, protein expression and purification

Preparation of plasmids, recombinant expression in *P. pastoris* and purification of pyranose dehydrogenase from *A. meleagris* (AmPDH) and variant H103Y were described in Sygmund et al. [17] and Krondorfer et al. [16].

### Enzymatic activity

PDH activity was determined using the standard assay with ferrocenium hexafluorophosphate and d-glucose at 30 °C as described before [18]. pH optima of AmPDH and variant H103Y were measured from pH 2.5–6 (100 mM citrate buffer), pH 6–8 (100 mM phosphate buffer) and pH 8–10 (100 mM borate buffer). Protein concentrations were determined using the method of Bradford with a pre-fabricated assay (BioRad, Hercules, CA) or photometrically with a U-3000 spectrophotometer (Hitachi, Tokyo, Japan) using the absorbance at 280 nm and the molar absorbance coefficients (*ε*_AmPDH_ = 67,840 M^−1^ cm^−1^; *ε*_H103YAmPDH_ = 69,330 M^−1^ cm^−1^).

### Electronic circular dichroism spectroscopy

Far UV electronic circular dichroism (ECD) spectra of the wild-type and the variant enzyme were recorded using a Chirascan CD Spectrophotometer (Applied Photophysics, Leatherhead, UK) in the wavelength range from 180 to 260 nm. The instrument was flushed with nitrogen, the pathlength was 1 mm, spectral bandwidth was set to 3 nm and the scan time per point to 10 s. The protein concentration of both samples was adjusted to 8 μM using 50 mM sodium phosphate buffer, pH 7.5.

### Electron paramagnetic resonance (EPR) spectroscopy

Electron paramagnetic resonance (EPR) measurements were carried out on a Bruker EMX continuous wave (CW) spectrometer, operating at X-band (9 GHz) frequencies, equipped with a high sensitivity resonator. EPR measurements were carried out using 50 μl of wild-type AmPDH (650 μM) or H103Y AmPDH (1000 μM). The solutions were transferred into 50 μl capillary tubes (Blaubrand, Wertheim, Germany) for EPR measurements at room temperature. EPR spectra were recorded using 2 mW microwave power (MWP), 100 kHz modulation frequency (MF), 0.5 mT modulation amplitude (MA), 41 ms conversion time (CT), 41 ms time constant (TC) and 1024 points. Simulations of EPR spectra were conducted using the software EasySpin [19] and *g*-values are expressed relative to diphenylpicrylhydrazyl (DPPH) (*g *= 2.0036), which was used as an external standard.

### Stopped-flow spectroscopy

Pre-steady-state kinetics of the oxidative and reductive half-reaction were studied using an Applied Photophysics SX20 (Leatherhead, UK) stopped-flow instrument with diode array and photo-multiplier (PMT) detection. All measurements were conducted in 65 mM sodium phosphate buffer, pH 7.5, using 20 μM of enzyme for the measurements with PMT and 40–60 μM for the diode array prescans in the single mixing mode. Reduced/oxidized enzyme was obtained using 1.2 equivalents of d-glucose/ferrocenium hexafluorophosphate as the wild-type AmPDH was present in the reduced state after purification and the variant in the oxidized state. For the oxidative half-reaction, different concentrations of ferrocenium were used (25–300 μM). Reduction of the FAD was studied by addition of d-glucose (0.1–5 mM). All concentrations are given after mixing. Time traces at 462 nm were fit to a single exponential function using the Pro Data Viewer Software (Applied Photophysics), standard deviation was calculated from at least 3 measurements. Obtained *k*_obs_-values were plotted against the substrate concentration and the apparent bimolecular rate constant (*k*_app_) was obtained from the slope.

### Spectroelectrochemistry

All experiments were carried out in a homemade optical transparent thin-layer spectrochemical (OTTLE) cell with a pathlength of 0.05 cm. The three-electrode configuration consisted of a platinum gauze working – (Goodfellow Cambridge Ltd., Huntington, England, UK) a platinum wire auxiliary – (Goodfellow Cambridge Ltd.) and a RE-6 Ag/AgCl-reference electrode (BASi, West Lafayette, IN, USA). The reference electrode was calibrated against a saturated calomel electrode (HgCl). All potentials are referenced to the standard hydrogen electrode (SHE, +242 mV). Potentials were applied across the OTTLE cell with a Series G 300 Potentiostat/Galvanostat/ZRA (Gamry, Warminster, PA, USA). Constant temperature of 25 °C was maintained by a Universal-Thermostat U200 (ELV Elektronik Ag, Leer, Germany) connected to a circulating F12-ED water bath (Julabo, Seelbach, Germany). UV–vis spectra were recorded with an Agilent 8453 UV–vis Diode Array System (Agilent Technologies, Santa Clara, CA, USA).

Spectroelectrochemical experiments were performed using 500 μl samples containing 1000 μM wild-type or mutant protein in 100 mM phosphate buffer, pH 7.4, plus 100 mM KCl, in the presence of various mediators: methylviologen (150 μM), anthraquinone-1,5-disulfonate, 2-hydroxy-1,4-naphtoquinone, indigo carmine, indigotrisulfonate, duroquinone, methylene blue, phenazine methosulfate, 1,2-naphtoquinone and N,N,N′,N′-tetramethyl-p-phenylenediamine (all 3 μM). Nernst plot consisted of at least seven points and were invariably linear with a slope consistent with a two-electron reduction process.

### Differential scanning calorimetry (DSC)

DSC measurements were conducted as described in Hofbauer et al. [20] using 16 μM of AmPDH and variant H103Y in 65 mM sodium phosphate buffer, pH 7.5. For data analysis and conversion the Microcal Origin 7.0 software was used. Heat capacity (*C*_p_) was expressed in kcal mol^−1^ K^−1^ (1 cal = 4.184 J). Data points were fitted to non-two-state equilibrium-unfolding models by the Lavenberg/Marquardt (LM) non-linear least square method.

#### *Thermo*FAD

Unfolding of the proteins was also followed by the increased emission of fluorescence of the FAD cofactor upon thermal denaturation [21]. Around 2 μl of concentrated protein were diluted with selected buffers to a final concentration of 16 μM in a final volume of 25 μl. Applied buffers were: 100 mM sodium acetate buffer, pH 3, 4 and 5; 100 mM sodium phosphate buffer pH 6, 7 and 8. The samples were heated in steps of 0.5 °C from 20 to 95 °C in a MyiQ Real-Time PCR cycler (BioRad) and fluorescence data were recorded. The *T*_m_ value was obtained as the maximum of the first derivative of the sigmoid curve [22] and represents the mean value of two independent experiments.

### Chemical denaturation followed by fluorescence spectroscopy

Guanidinium hydrochloride (GdnHCl) was used for chemical denaturation of the wild-type AmPDH and variant H103Y. The changes in intrinsic tryptophan emission were followed by fluorescence spectroscopy. 0.8 μM AmPDH and H103Y in 65 mM sodium phosphate buffer, pH 7.5, were incubated with increasing concentrations of GdnHCl (0–6.5 M) for 18 h at room temperature. The excitation wavelength was set to 295 nm, an emission wavelength scan was conducted with a PerkinElmer Enspire plate reader from 320 to 400 nm at room temperature. The fraction of unfolded protein *α* was calculated for each GdnHCl concentration according to *α* = (*F*_N_−*F*)/(*F*_N_−*F*_U_). *F*_N_ is the fluorescence emission maximum of the protein in the native, unfolded state, *F*_U_ the fluorescence emission maximum at the completely unfolded state. *F* represents the emission maximum at a defined GdnHCl concentration. The free enthalpy, Δ*G*^0^, was determined according to Δ*G*_0_ = −*RT*ln*K *= −*RT*ln[*α*/(1−*α*)] where *R* is the gas constant, *T* is the absolute temperature and *K* is the equilibrium constant. *m* is the slope of the linear curve $\Delta G^{0}=\Delta G_{{\text{H}}_{2}\text{O}}^{0}-(m*[\text{GdnHCl}])=m*(c_{m}-[\text{GdnHCl}])$, c*_m_* is the concentration of GdnHCl with *K *= 1.

### Long-term stability studies

Dilutions of AmPDH and variant H103Y in 65 mM sodium phosphate buffer were incubated in an Eppendorf tube at different temperatures and the remaining enzymatic activity was determined regularly over 35 weeks (4 °C, 21 °C, 30 °C) and 24 h (40 °C and 50 °C) in duplicates.

## Results and discussion

The mechanism of the formation and the impact of a covalent FAD-linkage on enzyme catalysis were subject of several studies [3]. However, the effects of the removal of the covalent bond are very diverse in the flavoproteins investigated so far. PDH is a flavoprotein dehydrogenase with a covalently bound FAD, which is rather rare. Therefore the impact of the disruption of the covalent bond on catalysis as well as on the conformational and thermal stability were studied in variant H103Y and compared to the wild-type protein.

### Overall structure and enzymatic activity

**Fig. 1:**
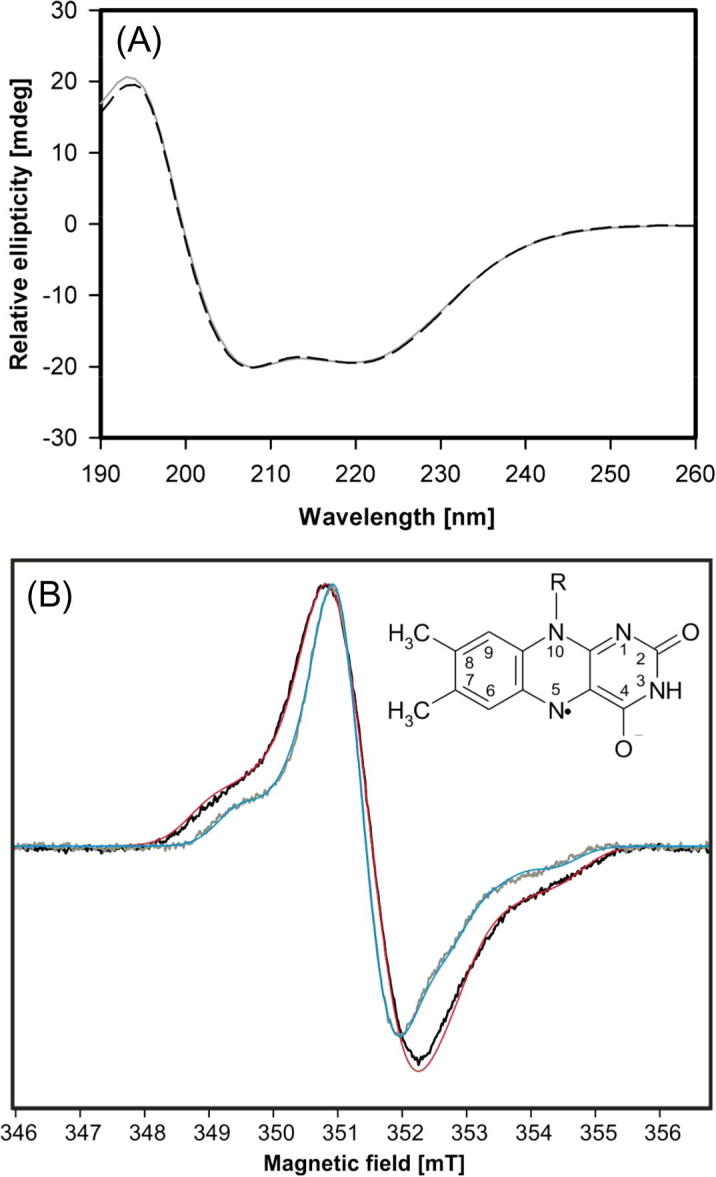
(A) Far-UV ECD spectra of 8 μM AmPDH (black) and H103YAmPDH (gray) in 65 mM sodium phosphate buffer pH 7.5. (B) Cw EPR spectra of wild-type AmPDH (black) and H103Y AmPDH variant (gray) and their simulations (red, blue) recorded at room temperature at pH 7.5. Simulation parameters for wild-type AmPDH (red) are *g*_iso_ = 2.0028, *A*_iso_ (N^5^) = 0.78 mT, *A*_iso_ (N^10^) = 0.32 mT and for H103Y AmPDH *g*_iso_ = 2.0028, *A*_iso_ (N^5^) = 0.63 mT, *A*_iso_ (N^10^) = 0.34 mT. (For interpretation of the references to color in this figure legend, the reader is referred to the web version of this article.)

Fig. 1A depicts the far-UV ECD spectra [23] of wild-type AmPDH and the variant H103Y. Both spectra are very similar showing two minima at 208 and 222 nm suggesting dominating α-helical structures, which nicely reflect the known crystal structure of AmPDH. The almost identical spectra of wild-type and variant protein clearly demonstrate that the exchange of histidine 103 had no effect on the overall fold and only provoked localized structural changes. Thus the covalent FAD to protein linkage is not a prerequisite for proper folding of PDH.

**Fig. 2:**
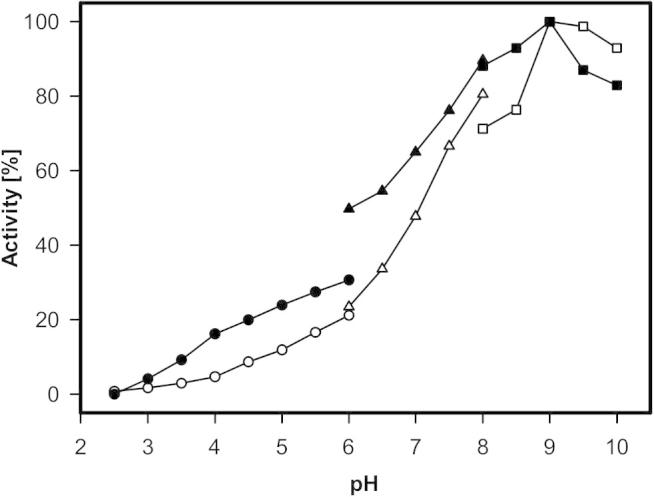
Effect of pH on the overall activity of AmPDH (black) and AmPDH variant H103Y (white) with ferrocenium hexafluorophosphate and 25 mM d-glucose at 30 °C. Circles: citrate buffer; triangles: phosphate buffer; squares: borate buffer.

The mutation of histidine 103 to tyrosine was previously shown to have a negative effect on steady-state kinetic parameters of AmPDH. The catalytic efficiencies towards sugar substrates and electron acceptors decreased substantially [16]. However, these findings could not be explained satisfactorily. This actually motivated us to the present study. In the beginning the pH dependence of PDH activity was tested using the standard assay with ferrocenium hexafluorophosphate and d-glucose at 30 °C and buffers from pH 2.5 to 10 (Fig. 2). Both AmPDH and variant H103Y exhibited maximum activity around pH 9. The wild-type enzyme showed higher relative activity than variant H103Y from pH 2.5 to 8.5. At pH > 8.5 the variant was more active. Interestingly, at pH 6 the wild-type flavoprotein shows about 20% higher activity in sodium phosphate buffer compared to citrate buffer, whereas there was no impact on the variant.

Since the variant H103Y is still catalytically active, it must (non-covalently) bind a redox-active FAD-cofactor. This has been further confirmed by the stopped-flow and EPR measurements shown below and allows the conclusion that the mutation did not cause drastic alterations of the active site geometry (which was also suggested by the ECD data). In the closely related GMC-member POx, the overall crystal structure as well as the active site of variant H167A was found to be nearly identical to the wild-type [15]. A similar result was obtained for the flavoprotein vanillyl-alcohol oxidase (VAO). A comparison of the crystal structure of variant H422A with the wild-type gave a root mean square deviation for all C-α atoms of only 0.27 Å. The active site architecture was similar to the wild-type, even an identical substrate-binding-mode could be observed [24]. In contrast to that finding, variant H46A in alditol oxidase, also member of the VAO family of flavoproteins, was expressed in a partially insoluble state and did not bind FAD at all. Therefore the authors supposed that folding and the (active site) structure were impaired [25]. Chito-oligosaccharide oxidase from *Fusarium graminearum* contains a double-covalent FAD-linkage. A disruption of both covalent bonds (H94A/C154A) drastically diminished the expression levels, supporting the role of covalent flavin linkages in protein stability. The expression of the single variants yielded similar expression levels as the wild-type [26]. These observations of representatives of different superfamilies do not allow to distinguish a clear trend or a reason for the structural integrity determinants of these non-covalent flavoprotein variants. Factors that have a large influence are the expression host, the geometry of the (substituted) amino acid that covalently binds the FAD in the active site and the choice of the replacing amino acid.

### Oxidative and reductive half reaction

To identify the rate-limiting step in the reaction catalyzed by PDH, stopped-flow experiments of the reductive and oxidative half-reaction of the wild-type and variant H103Y at 30 °C were conducted. For a first impression of the changes occurring, spectral scans using diode array detection were performed. For calculation of bimolecular rate constants measurements at 462 nm were performed.

**Fig. 3:**
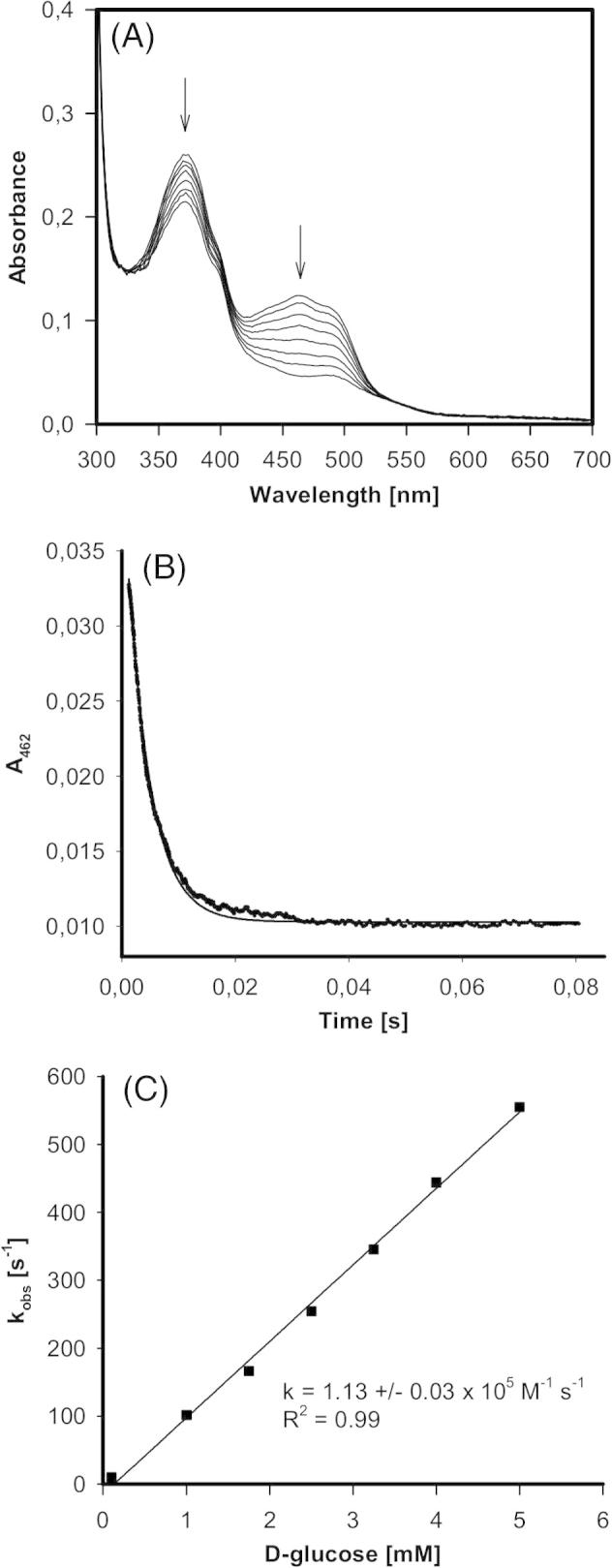
Reductive half-reaction of wild-type AmPDH followed by stopped-flow spectroscopy. (A) Spectral changes upon reduction of 60 μM AmPDH with 200 μM d-glucose at 30 °C. (B) Typical time trace and fit at 462 nm of the reaction between 21 μM AmPDH and 2.5 mM d-glucose at 30 °C. (C) Plot of the pseudo-first-order rate constants *versus*d-glucose concentration.

Fig. 3A depicts the spectral changes of wild-type AmPDH upon addition of d-glucose. It was interesting to see that the purified recombinant protein existed in a mixed oxidation state (FAD/FADH_2_) (compare with Fig. 5A). During the reaction the absorbance around the maxima at 372 nm and 462 nm rapidly decreased (Fig. 3A). The reaction was monophasic and the time traces at 462 nm could be fitted by a single-exponential function (Fig. 3B). Upon plotting the resulting *k*_obs_-values *versus* the glucose concentration an apparent bimolecular rate constant of (1.1 ± 0.03) × 10^5^ M^−1^ s^−1^ could be calculated.

**Fig. 4:**
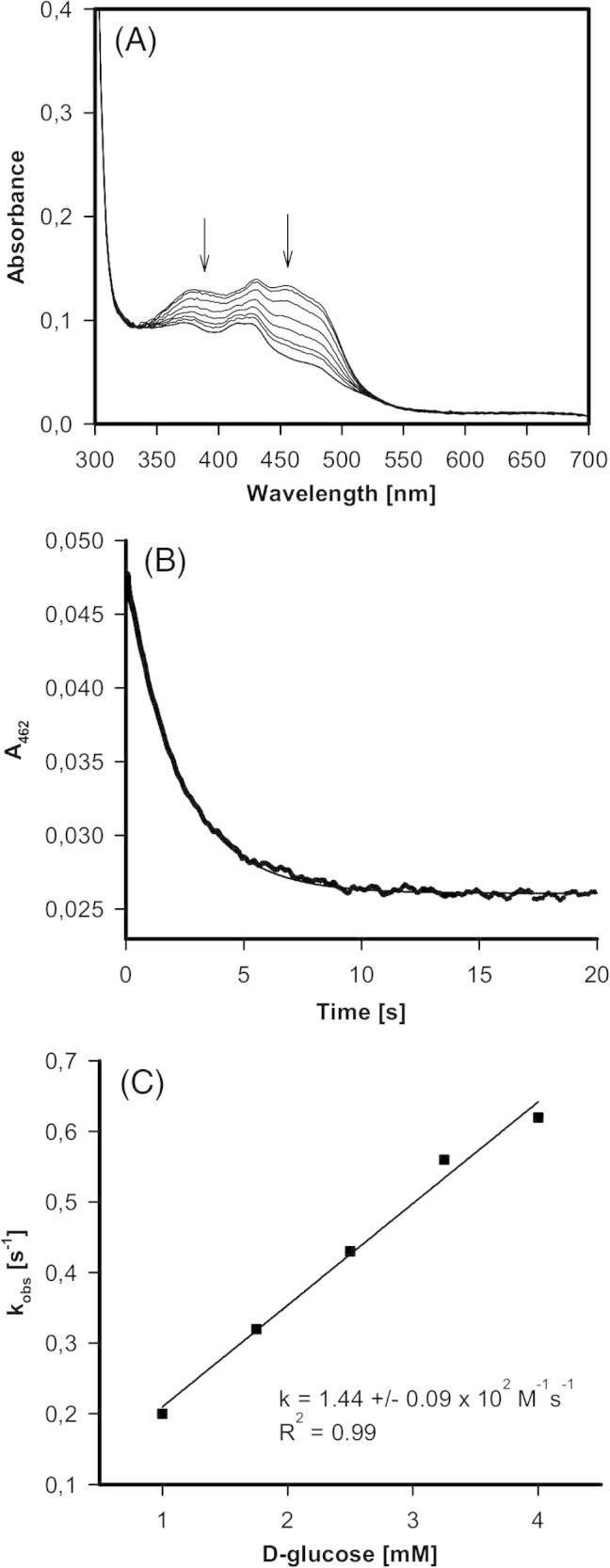
Reductive half-reaction of AmPDH variant H103Y followed by stopped-flow spectroscopy. (A) Spectral changes upon reduction of 40 μM variant with 25 mM d-glucose at 30 °C. (B) Typical time trace and fit at 462 nm of the reaction between 21 μM AmPDH and 2.5 mM d-glucose at 30 °C. (C) Plot of the pseudo-first-order rate constants *versus*d-glucose concentration.

**Fig. 5:**
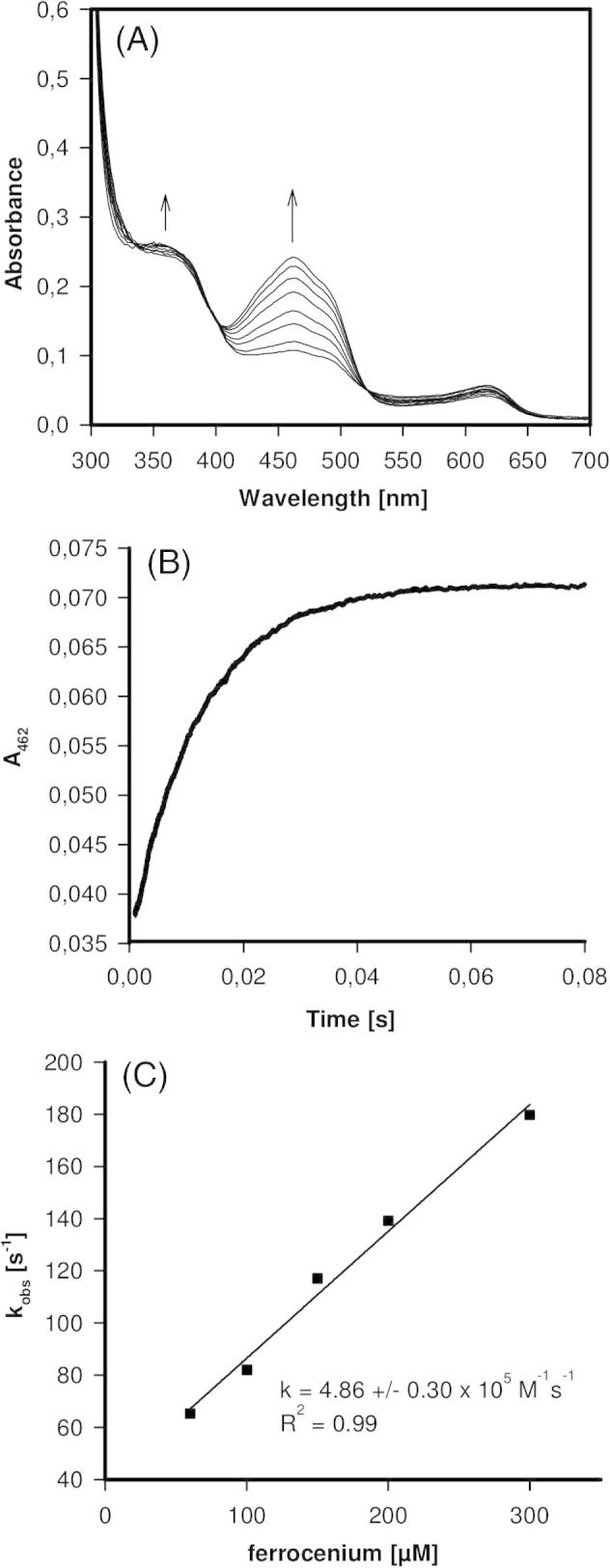
Oxidative half-reaction of AmPDH followed by stopped-flow spectroscopy. (A) Spectral changes of oxidation of 60 μM AmPDH by 200 μM ferrocenium hexafluorophosphate at 30 °C. (B) Typical time trace and fit at 462 nm of the reaction between 21 μM AmPDH with 100 μM ferrocenium hexafluorophosphate at 30 °C. (C) Plot of pseudo-first-order rate constants *versus* ferrocenium concentration.

Performing the same experiment with the H103Y variant demonstrated that the mutation significantly decelerated the two-electron transfer reaction from glucose to the redox cofactor (Fig. 4). The UV–vis spectrum of the purified recombinant variant was different from that of the wild-type protein. The two maxima typical for a flavoprotein were superimposed by a third maximum around 430 nm which might suggest the presence of a semiquinone radical (see below). Nevertheless, there was a clear monophasic reduction of the cofactor within the first 10 s (Fig. 4B) that strictly depended on the sugar concentration. The reaction was about 800-times slower compared to the wild-type enzyme (i.e. *k*_app_ = (1.4 ± 0.09) × 10^2^ M^−1^ s^−1^). With both enzymes there was an additional small and very slow reduction of FAD cofactor when monitored at longer time frame, however this phase was independent of the glucose concentration (not shown).

**Fig. 6:**
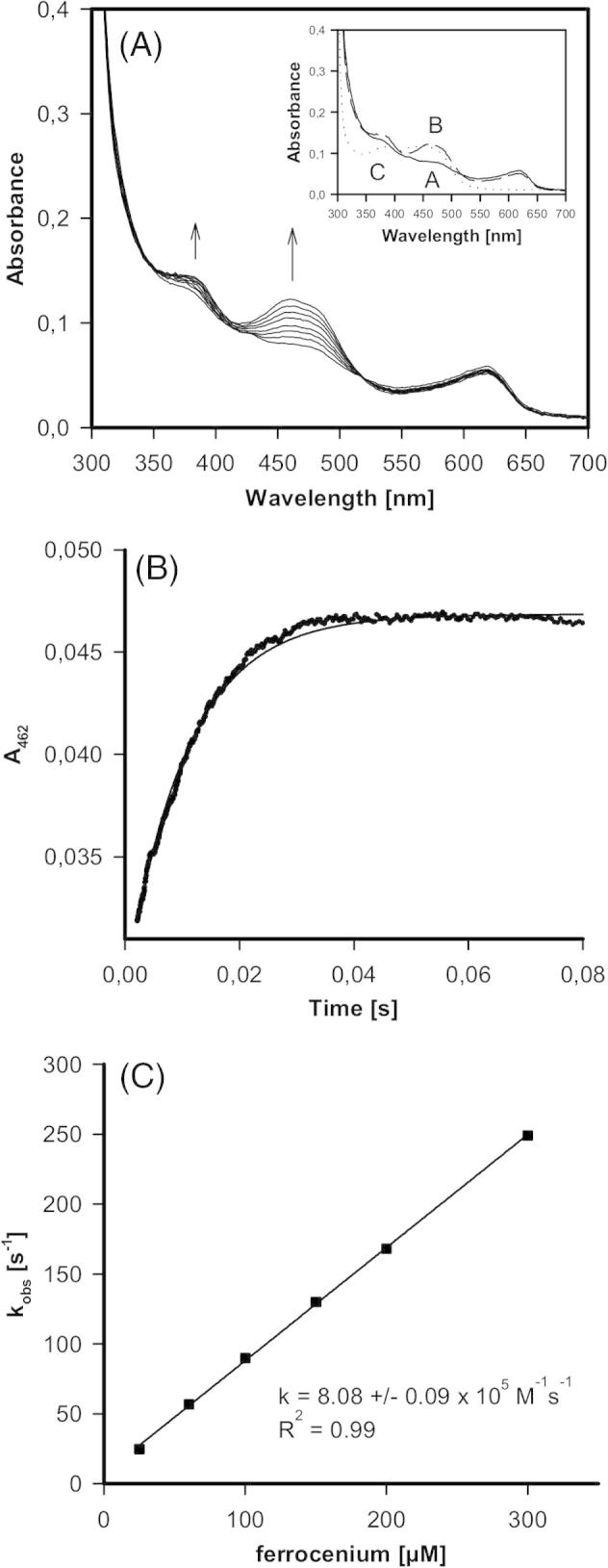
Oxidative half-reaction of AmPDH variant H103Y followed by stopped-flow spectroscopy. (A) Spectral changes of oxidation of 60 μM AmPDH variant H103Y by 200 μM ferrocenium hexafluorophosphate at 30 °C. Inset: reduced state (a), oxidized state (b) and spectrum observed after prolonged measurement of 50 s (c). (B) Typical time trace and fit at 462 nm of the reaction between 21 μM AmPDH variant H103Y with 100 μM ferrocenium hexafluorophosphate at 30 °C. (C) Plot of pseudo-first-order rate constants *versus* ferrocenium concentration.

For studying the oxidative half reaction (i.e. reoxidation of the redox cofactor by an artificial electron acceptor) ferrocenium hexafluorophosphate was used. In contrast to the reductive half-reaction the kinetics of reoxidation of FADH_2_ by the artificial electron acceptor was similar (Fig. 5, Fig. 6). There was a clear linear dependence of the *k*_obs_-values of absorbance increase at 462 nm from the concentration of ferrocenium hexafluorophosphate. The calculated apparent bimolecular rate constants were (4.9 ± 0.3) × 10^5^ M^−1^ s^−1^ (wild-type) and (8.1 ± 0.1) × 10^5^ M^−1^ s^−1^ (H103Y). During this transition the absorbance increase at 462 nm was more pronounced than that at 360 nm (Fig. 5A).

This monophasic fast reoxidation reaction was followed by a further slow transition. After prolonged reaction time in the variant H103Y the absorbance at 462 nm was blue-shifted and a peak at 430 nm emerged (see inset to Fig. 6A, spectrum C). This spectrum resembles the first spectrum observed during reduction of variant H103Y by d-glucose (Fig. 4A) and might suggest the formation of a semiquinone state of the prosthetic group. This intermediate cannot be physiologically relevant as it emerges after several seconds of incubation. A neutral semiquinone species is reported to exhibit absorbance in the range of 580–620 nm, whereas the anionic form shows characteristic absorbance at 380 nm and around 400 nm [27]. The appearance of such a spectral intermediate (anionic semiquinone) was reported for wild-type AmPDH, indicated by shoulders around 395 and 495 nm [4]. It has to be mentioned that maxima at 625 nm and around 330–350 nm are due to the absorbance of the electron acceptor ferrocenium hexafluorophosphate.

The presented presteady-state investigations clearly demonstrated that the reductive half reaction is the main determinant for the catalytic efficiency of AmPDH. The reduction of the FAD was slowed down by about three orders of magnitude in the non-covalent FAD-bound variant H103Y compared to wild-type PDH. One proposed function of the covalent FAD-bond is the maintenance of a high redox potential, as for non-covalently bound flavoproteins a much lower midpoint potential was observed [3]. Non-covalent variants like H167A in POx [15], H422A in VAO [24] or H69A in cholesterol oxidase [28] showed a decrease in redox potential due to the amino acid replacement. A lower redox potential is accompanied by a decrease of the bimolecular rate of the reductive half reaction, as observed here for PDH variant H103Y.

**Supplementary Fig. 1:**
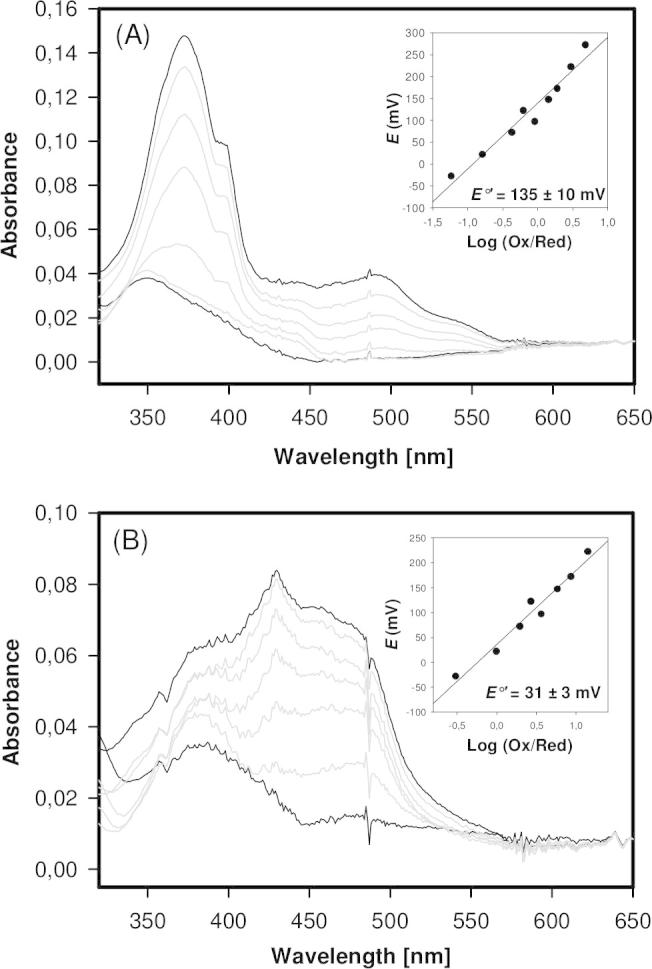
Spectroelectrochemistry of wild-type AmPDH (A) and the variant 573 H103Y (B) showing representative electronic spectra at various potentials at 25 °C and pH 574 7.4. The insets depict the corresponding Nernst plots.

In order to investigate differences in the standard reduction potential between wild-type AmPDH and the variant H103Y spectroelectrochemical measurements were performed. Supplemental Fig. 1 depicts a representative family of spectra of the wild-type protein and the variant at different applied potentials in the OTTLE cell. The calculated standard reduction potential determined from the corresponding Nernst plot (inset to Supplemental Fig. 1), was calculated to be +135 ± 10 mV and +31 ± 3 mV, respectively, at 25 °C and pH 7.4. The slope of the Nernst plot indicates that two electrons are exchanged. This finding clearly demonstrates that the covalent FAD attachment modulates the redox properties of AmPDH and flavoproteins in general [3].

Surprisingly, the oxidative half-reaction occurs nearly two times faster in variant H103Y than in the wild-type using ferrocenium hexafluorophosphate as the electron acceptor. A fivefold increase in oxygen reactivity was recently reported for this variant [16]. In VAO, the same effect was observed. Flavin reduction by electron donors was decreased and the oxidative half-reaction occurred slightly faster in variant H422A than in wild-type VAO [24].

### Semiquinone radical formation

As outlined above there was evidence from UV–vis spectroscopy for the existence of a stable semiquinone radical in the resting state of H103Y variant of AmPDH (Fig. 4A and inset to Fig. 6A). This prompted us to use electron paramagnetic resonance (EPR) spectroscopy for identification of this paramagnetic species. Interestingly, both proteins (wild-type and variant) unequivocally have a semiquinone radical in their stable resting state at pH 7.5.

The EPR spectrum of wild-type AmPDH is characterized by a peak of linewidth Δ*B*_PP_ = 1.43 mT and two shoulders at lower and higher magnetic field, separated by about 5 mT (Fig. 1B, black line). This spectrum is characteristic for an anionic semiquinone radical representing the interaction of the unpaired electron with two ^14^N nitrogen atoms at position 5 and 10 in FAD (with the shoulders originating from the *A*_zz_ value of the ^14^N coupling) [29]. The presence of the semiquinone anion radical in the resting state of wild-type AmPDH indicates an equilibrium of the oxidized form of the enzyme with its reduced forms.

The variant H103Y AmPDH showed a similar EPR spectrum but with a smaller linewidth of Δ*B*_PP_ = 1.06 mT and adjacent shoulder peaks separated by ∼4.5 mT (Fig. 1B, gray line). The origin of this radical is also an anionic semiquinone indicating a higher mobility of the FAD due to a smaller linewidth. This correlates with the non-covalently bound FAD in the H103Y variant and shows an equilibrium of the reduced enzyme with its oxidized forms. Simulations of both EPR spectra are shown in Fig. 1B (red and blue line).

### Thermal and conformational stability

**Fig. 7:**
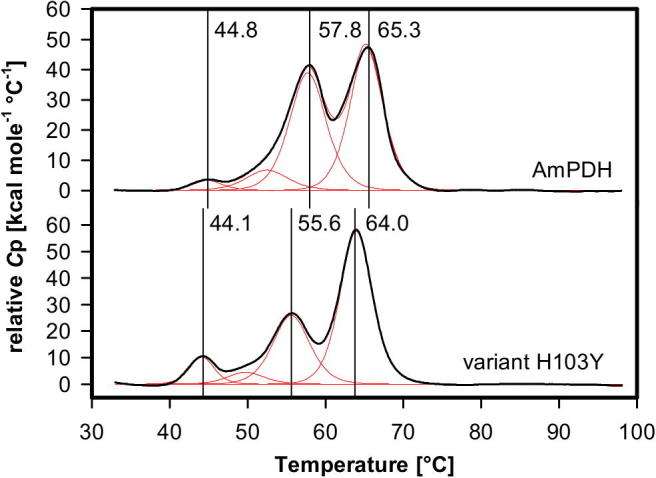
Thermostability of AmPDH and AmPDH variant H103Y determined by differential scanning calorimetry in 65 mM sodium phosphate buffer pH 7.5. Black lines: original data. Red lines: fits of endotherms to a non-two-state transition model.

Next we investigated the role of the FAD-protein linkage on the thermal and conformational stability. The thermostability of AmPDH and variant H103Y (in 65 mM sodium phosphate buffer pH 7.5) was investigated using differential scanning calorimetry (DSC). Fig. 7 shows the thermograms of the two enzymes which show non-two-state unfolding for both the wild-type and the variant. The wild-type enzyme shows two main transitions with *T*_m_-values at 57.8 and 65.3 °C, respectively, which might reflect the consecutive melting of the two domains (i.e. a substrate binding domain and a cofactor binding domain [4]) of this flavoprotein. In the variant both transitions occur at lower temperatures (55.6 and 64 °C). In addition in both proteins there is a third smaller endotherm at 44.8 (44.1 °C) which might reflect the unfolding of present apoprotein. The endotherm is more pronounced in the variant. The loss of the FAD cofactor is more likely to occur in the non-covalent variant than in the wild-type, which is also reflected by the calculated calorimetric enthalpies (areas under fitted curves) of the respective transitions. In wild-type PDH the calorimetric enthalpy for the first transition (11.4 ± 2.0 kcal/mol) reflecting the melting of the apoprotein is significantly smaller than the enthalpy for the corresponding transition in variant H103Y (43.5 ± 1.3 kcal/mol). By contrast, the calculated enthalpy of the second transition at around 56 °C in wild-type PDH (201.4 ± 8.9 kcal) is significantly larger than the enthalpy for the respective transition in variant H103Y (163.4 ± 2.6 kcal), which might suggest that this transition reflects the unfolding of the FAD-binding domain. Taking into account the error in calculation of enthalpies, the observed differences between wild-type protein and variant in the transitions at 44 °C and 56 °C nicely compensate each other (Δ*H*_apo_ = 32.1 ± 3.3 kcal; Δ*H*_FAD-domain_ = 38.0 ± 11.5 kcal).

**Fig. 8:**
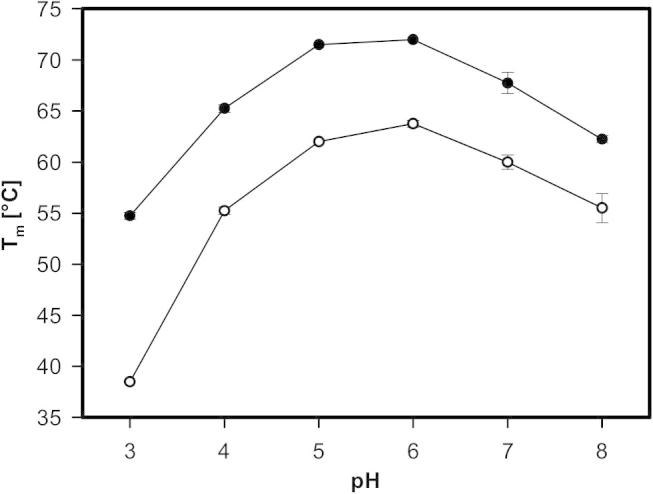
Plot of *T*_m_ values of wild-type AmPDH (black) and variant H103Y (white) determined by *Thermo*FAD at different pH values in 100 mM sodium acetate buffer (pH 3–5) or 100 mM sodium phosphate buffer (pH 6–8).

In order to assess the influence of pH on thermostability, *Thermo*FAD experiments using buffers with different pH were carried out. *Thermo*FAD was first described by Forneris et al. [21]. In this method, the intrinsic fluorescence of the FAD cofactor is used to assess the thermostability of a protein. The technique can be applied to flavoproteins with covalently as well as non-covalently attached flavin. In the native, folded state the fluorescence is quenched by the surrounding protein environment. With increasing temperature, the protein starts to unfold, the FAD is exposed to the solvent and the increase in fluorescence can be detected. The concentrated protein samples were diluted in sodium acetate or phosphate buffer (pH 3–8) and the fluorescence was measured at increasing temperatures. The determined *T*_m_ values were highly reproducible and revealed that both enzymes are most thermostable at pH 6 in sodium phosphate buffer with *T*_m_ values of 72 °C for the wild-type and 63.8 °C for variant H103Y (Fig. 8). The difference in *T*_m_ between the wild-type and the variant decreases with increasing pH. At acidic pH values the variant is more destabilized than the wild-type protein. One might speculate whether the influence of pH on thermostability can be attributed to the protonation state of the histidine 103 in the wild-type which was replaced by a tyrosine in the variant.

Additionally, AmPDH and variant H103Y were incubated over several months to probe the long-term stability at 4 °C, room temperature (21 °C) and 30 °C in 65 mM sodium phosphate buffer. Residual activity was determined using the standard ferrocenium/d-glucose assay. The stability of the wild-type protein decreased with increasing incubation temperature, after 35 weeks 77%, 46% and 21%, respectively, of the activity remained. The variant H103Y showed a different behavior, 35 weeks of incubation at 4 °C increased the activity to 152%, whereas the residual activities after incubation at 21 °C and 30 °C were nearly identical with 27% and 28%.

**Fig. 9:**
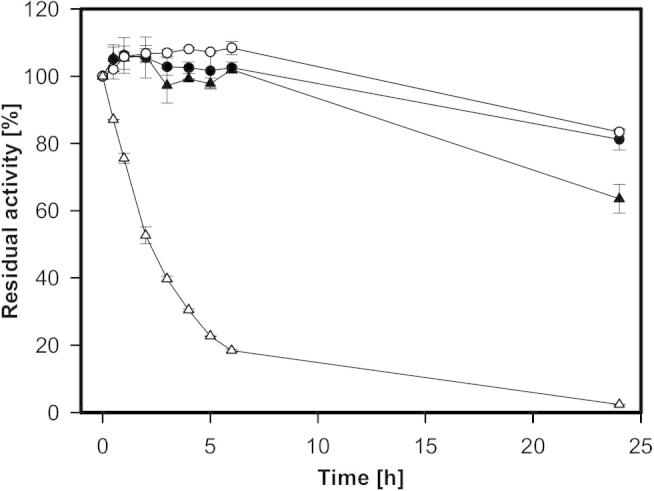
Residual activity of AmPDH (black) and AmPDH variant H103Y (white) after incubation at 40 °C (circles) and 50 °C (triangles) in 65 mM sodium phosphate buffer pH 7.5. Activity was determined using the standard ferrocenium/d-glucose assay.

The temperature stability at 40 °C and 50 °C was investigated over 24 h. AmPDH and variant H103Y show similar stability at 40 °C with 81% and 83% residual activity, the activity dropped slowly for both enzymes (Fig. 9). At 50 °C the wild-type protein is much more stable than the variant with 63% *versus* 2% residual activity after 24 h. Variant H103Y already reached its half-life after around 2.5 h of incubation, whereas wild-type AmPDH slowly lost activity.

In general, PDH is a very stable enzyme, the half-life at 4 °C is longer than one year. For the wild-type, the residual activity decreased gradually with increasing incubation temperature whereas the variant showed a harsh drop of the residual activity. The increase in activity of variant H103Y at 4 °C could be due to a loss of FAD cofactor upon freezing (after purification) and thawing incubation.

**Fig. 10:**
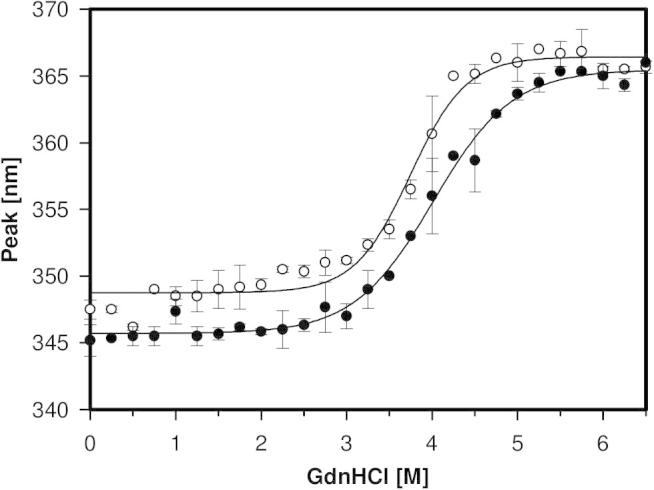
GdnHCl mediated unfolding of AmPDH (black) and AmPDH variant H103Y (white) followed by fluorescence spectroscopy (excitation 295 nm). 800 nM of protein was incubated around 18 h with GdnHCl concentrations from 0 to 6.5 M in 65 mM sodium phosphate buffer pH 7.5. The fluorescence emission maximum was plotted against the GdnHCl concentrations.

To study the conformational stability of PDH chemical unfolding with guanidine hydrochloride (GdnHCl) was performed monitoring changes of the intrinsic tryptophan fluorescence. Fig. 10 shows the shift of the fluorescence emission maximum after incubation of wild-type AmPDH and the variant H103Y with increasing concentrations of GdnHCl. Unfolding started around 2 M GdnHCl and was completed around 5 M for the variant and above 6 M for the wild-type protein. At first sight the obtained plots suggest a two-state unfolding, however with a broad transition range. Upon closer inspection it is evident that from 2.0 to 3.5 M GdnHCl a first unfolding event took place, which is more pronounced in variant H103Y. The variant H103Y showed a higher emission maximum at 0 M GdnHCl than the wild-type. This was also observed for variant H69A in cholesterol oxidase [30].

From the plots of the change in free enthalpy (Δ*G*^0^) *versus* the GdnHCl concentrations, $\Delta G_{{\text{H}}_{2}\text{O}}^{0}$ values of 19.25 ± 1.65 kJ mol^−1^ (wild-type protein) and 14.63 ± 0.24 kJ mol^−1^ (variant) were calculated (Table 1). Thus, besides having a higher thermal stability, also the conformational stability of the wild-type protein is higher. This is also reflected by a higher *c_m_* value (i.e. denaturant concentration required for midterm unfolding) in the wild-type protein (4.0 ± 0.07 M *versus* 3.7 ± 0.04 M). From the slope of the linear curve $\Delta G^{0}=\Delta G_{{\text{H}}_{2}\text{O}}^{0}-(m*[\text{GdnHCl}])$, *m* was calculated to be 4.8 ± 0.3 kJ mol^−1^ M^−1^ and 3.9 ± 0.0 kJ mol^−1^ M^−1^. The *m*-value stands for the effectivity of the denaturant and is proportional to the number of groups in a protein. A single mutation of two otherwise identical proteins should not influence the *m*-value. However, in the present case the covalent attachment of the cofactor is affected. This could explain that the single mutation has significant impact on the overall protein stability. PDH possesses eight tryptophans out of 577 amino acids, mainly located in the outer shell of the protein. Therefore the changes in tryptophan fluorescence are maybe not fully indicative for global changes in protein structure, but a clear destabilizing effect of the non-covalently linked FAD cofactor in variant H103Y could be observed.

**Table 1:** Thermodynamic parameters for GdnHCl-mediated unfolding followed by monitoring the intrinsic fluorescence of tryptophan (excitation 295 nm, emission 320–400 nm).

Thermodynamic parameter	AmPDH	AmPDH variant H103Y
$\Delta G_{{\text{H}}_{2}\text{O}}^{0}$ [kJ mol^−1^]	19.25 ± 1.65	14.63 ± 0.24
*m* [kJ mol^−1^ M^−1^]	4.80 ± 0.29	3.89 ± 0.00
*c_m_* [M]	4.01 ± 0.07	3.74 ± 0.04

## Summary and conclusions

In summary, it could be shown that the disruption of the FAD to protein covalent linkage does not impair the binding of the cofactor to the protein nor alter its overall secondary structure composition, but decreases the standard reduction potential. As a consequence the reductive half-reaction was about three orders of magnitude slower in the variant compared to the wild-type protein, whereas the impact on the oxidative half-reaction was relatively small. EPR spectroscopy unequivocally demonstrated the presence of semiquinone radicals in the resting state of both recombinant proteins and suggested a higher mobility of the cofactor in the variant. Moreover, disruption of the covalent linkage decreases the thermal and conformational stability of the protein.

In conclusion, the observed effects on catalysis can mostly be attributed to a decrease in the reduction potential of the cofactor and as a consequence in the decrease of the capacity of the variant to oxidize glucose. In the wild-type protein the cofactor is tightly bound to the protein and supports the structural integrity and stability of PDH.

## Footnotes

1 *Abbreviations used:* PDH, pyranose dehydrogenase; GMC, glucose–methanol–choline oxidase; EPR, electron paramagnetic resonance; DCIP, 2,6-dichloroindophenol; POx, pyranose 2-oxidase; VAO, vanillyl-alcohol-oxidase; MSOX, monomeric sarcosine oxidase; ECD, electronic circular dichroism; DSC, differential scanning calorimetry; CW, continuous wave; MWP, microwave power; MF, modulation frequency; MA, modulation amplitude; CT, conversion time; TC, time constant; DPPH, diphenylpicrylhydrazyl; PMT, photo-multiplier; OTTLE, optical transparent thin-layer spectrochemical; *C*_p,_ Heat capacity; GdnHCl, Guanidinium hydrochloride.
